# Combination of dirty mass volume and APACHE II score predicts mortality in patients with colorectal perforation

**DOI:** 10.1186/s13017-021-00359-y

**Published:** 2021-03-30

**Authors:** Daichi Ishikawa, Yukako Takehara, Atsushi Takata, Kazuhito Takamura, Hirohiko Sato

**Affiliations:** Department of Surgery, Yoshinogawa Medical Center, 120 Nishichiejima, Yoshinogawa City, Tokushima 776-8511 Japan

**Keywords:** Colorectal perforation, Dirty mass, APACHE II, Prognostic factor

## Abstract

**Background:**

“Dirty mass” is a specific computed tomography (CT) finding that is seen frequently in colorectal perforation. The prognostic significance of this finding for mortality is unclear.

**Methods:**

Fifty-eight consecutive patients with colorectal perforation who underwent emergency surgery were retrospectively reviewed in the study. Dirty mass identified on multi-detector row CT (MDCT) was 3D-reconstructed and its volume was calculated using Ziostation software. Dirty mass volume and other clinical characteristics were compared between survivor (*n* = 45) and mortality groups (*n* = 13) to identify predictive factors for mortality. Mann–Whitney *U* test and *Χ*^2^ test were used in univariate analysis and logistic regression analysis was used in multivariate analysis.

**Results:**

Dirty mass was identified in 36/58 patients (62.1%) and located next to perforated colorectum in all cases. Receiver-operating characteristic (ROC) curve analysis identified the highest peak at 96.3 cm^3^, with sensitivity of 0.643 and specificity of 0.864. Univariate analysis revealed dirty mass volume, acute disseminated intravascular coagulation (DIC) score, acute physiology and chronic health evaluation II (APACHE II) score, and sequential organ failure assessment (SOFA) score as prognostic markers for mortality (*p*<0.01). Multivariate analysis revealed dirty mass volume and APACHE II score as independent prognostic indicators for mortality. Mortality was stratified by dividing patients into four groups according to dirty mass volume and APACHE II score.

**Conclusions:**

The combination of dirty mass volume and APACHE II score could stratify the postoperative mortality risk in patients with colorectal perforation. According to the risk stratification, surgeons might be able to decide the surgical procedures and intensity of postoperative management.

## Introduction

Colorectal perforation is an emergent status that causes severe sepsis, disseminated intravascular coagulation (DIC), and multiple organ failure [1]. Despite recent advances in surgical techniques and intensive perioperative care, mortality rates remain high [2, 3]. Thus, it is important to predict postoperative outcome by conducting preoperative examinations and evaluating the patient’s condition. Predictive factors include such as older age, low preoperative blood pressure, arterial blood lactate concentration as well as scoring systems such as acute physiology and chronic health evaluation II (APACHE II) score, sequential organ failure assessment (SOFA) score, and physiological and operative severity for the enumeration of mortality and morbidity (POSSUM) score [4–7].

Multidetector computed tomography (MDCT) is a rapid imaging modality that is commonly used in the setting of acute abdominal emergency, and is the most reliable modality for diagnosing colorectal perforation [8]. Focal collection of extraluminal fecal matter is a specific finding of colorectal perforation that can be identified on MDCT. Saeki et al. first reported extraluminal stool as “dirty mass” and described its characteristics [9]. However, the significance of dirty mass on the severity of colorectal perforation and on patient prognosis has not been elucidated. The aims of the present study were to quantify dirty mass volume and investigate its association with mortality, together with other previously reported prognostic factors and scoring systems.

## Materials and methods

### Patients

Fifty-eight consecutive patients with colorectal perforation who underwent emergency surgery at the Department of Surgery in Yoshinogawa Medical Center between 2012 and 2020 were enrolled in the study. Patients with perforation of the appendix or iatrogenic perforation were excluded from the study. Patients’ clinical data, including medical history, physical examination and preoperative laboratory data, and details of treatments were retrospectively reviewed from the medical records. The patients’ characteristics are listed in Table 1. At 30 days after emergency surgery, 45 patients (77.6%) survived and 13 (22.4%) had died. The patients were divided into a survivor group and a mortality group for further analysis. Patient characteristics and therapeutic factors were compared between the survivor and mortality groups (Tables 2 and 3). Scoring systems such as the acute DIC score established by Japanese Association for Acute Medicine, and APACHE II, SOFA, and POSSUM scores were calculated preoperatively.

**Table 1:** Patient characteristics

Factor	(*n*=58)
Age (years)	80 (36-94)
Male	29 (50)
BMI	21.7 ± 3.8
ASA-PS	
I	6 (10.3)
II	28 (48.3)
III	22 (37.9)
IV	2 (3.4)
Survived/mortality	45 (77.6)/13 (22.4)
Comorbidity	38 (65.5)
Steroid	6 (10.3)
Cause	
Diverticulitis	24 (41.4)
Cancer	18 (31.0)
Ischemia	6 (10.3)
Idiopathic	5 (8.6)
Other	5 (8.6)
Perforation site	
Ascending colon	9 (15.5)
Transverse colon	7 (12.1)
Descending colon	4 (6.9)
Sigmoid colon	28 (48.3)
Rectum	10 (17.2)
Length of operation (min)	114 (54-182)
Blood loss (ml)	59 (10-1500)

Data are expressed as the median (range), mean ± standard deviation, or *n* (%)

*BMI* body mass index, *ASA-PS* American Society of Anesthesiologists-Physical Status

**Table 2:** Comparison of patient background, physical examination, and disease factors between the survivor and mortality groups

	Survivors	Mortality	*p* value
	(*n*=45)	(*n*=13)	
Age (years)	79 (36-94)	82 (68-90)	0.042^*^
Male	23	6	1.000
BMI (kg/m^2^)	21.9 ± 4.0	21.1 ± 2.4	0.632
ASA-PS			
I	6	0	
II	25	3	
III	13	9	
IV	1	1	0.028^*^
Comorbidity	26	12	0.048^*^
Steroid use	4	2	0.873
Heart rate (/min)	94 (56-134)	100 (54-143)	0.081
MAP (mmHg)	88 ± 17	75 ± 20	0.023^*^
Body temperature (°C)	37.3 (35.0-39.8)	36.9 (35.5-38.5)	0.102
Time from onset to surgery (h)			
>24 h	18	2	0.189
Cause			
Diverticulitis	3	21	
Cancer	6	12	
Idiopathic	0	5	
Ischemia	2	4	
Other	2	3	0.255
Perforation site			
Ascending colon	6	3	
Transverse colon	6	1	
Descending colon	3	1	
Sigmoid colon	22	6	
Rectum	8	2	0.922

Data are expressed as the median (range), mean ± standard deviation

Comorbidities include diabetes mellitus, heart, lung, or renal disease

*BMI* body mass index, *ASA-PS* American Society of Anesthesiologists-Physical Status, *MAP* mean arterial pressure

^*^p < 0.05

**Table 3:** Comparison of therapeutic factors, examination data, and score values between the survivor and mortality groups

	Survivors	Mortality	*p* value
	(*n*=45)	(*n*=13)	
Procedure			
Hartmann’s procedure	30	12	
Colectomy and PA	11	1	
Stoma creation	4	0	0.179
Length of operation	118 (54-182)	106 (72-152)	0.837
Blood loss	60 (10-1500)	59 (10-324)	0.747
Transfusion	20	5	0.948
Ventilator use	16	10	0.020^*^
PMX-DHP use	15	7	0.309
IVIG use	12	7	0.133
Thrombomodulin use	18	10	0.042^*^
Hemoglobin (g/dl)	12.1 ± 2.5	12.5 ± 2.8	0.608
WBC (/μl)	8660 (660-27940)	6100 (1240-15940)	0.531
Platelet (×10^3^/μl)	249 ± 120	219 ± 100	0.439
CRP (mg/dl)	12.3 ±11.8	6.4 ± 8.3	0.156
Albumin (mg/dl)	3.3 ± 0.7	3.0 ± 0.6	0.167
Creatinine (mg/dl)	1.5 ± 1.6	2.4 ± 2.2	0.060
Dirty mass volume (cm^3^)	54 ± 100	234 ± 211	0.004^**^
DIC score	1 (0-6)	3 (0-8)	0.007^**^
APACHE II score	11 (3-22)	18 (8-30)	0.001^**^
SOFA score	2 (0-6)	5 (0-12)	0.002^**^
POSSUM score	44 (29-64)	53 (34-74)	0.030^*^

Data are expressed as the median (range), mean ± standard deviation

*PA* primary anastomosis, *PMX-DHP* polymyxin B-immobilized fiber direct hemoperfusion, *IVIG* intravenous immunoglobulin, *WBC* white blood cells, *CRP* C-reactive protein, *DIC* disseminated intravascular coagulation, *APACHE II* acute physiology and chronic health evaluation, *SOFA* sequential organ failure assessment, *POSSUM* physiological and operative severity score for the enumeration of mortality and morbidity

^*^p < 0.05, ^**^p < 0.01

### Reconstruction and quantification of dirty mass

**Fig. 1:**
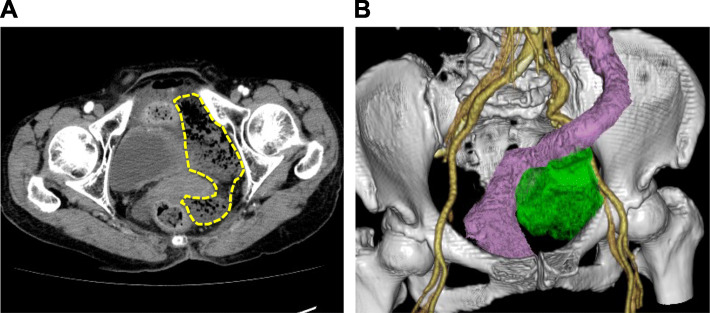
3D reconstruction and quantification of dirty mass with the Ziostation software. **a** The position of a dirty mass is indicated by the yellow-dashed line that was hand-traced on an axial MDCT image. These outlines were used to construct a 3D image. **b** Representative 3D image of a dirty mass constructed using Ziostation. The dirty mass (green) is located next to the sigmoid colon (pink). The volume of the dirty mass was calculated as 419.2 cm^3^

All patients underwent MDCT prior to emergency surgery and the images were assessed for the presence of colorectal perforation and dirty mass by two radiologists. Dirty mass was defined as a localized area of low attenuation containing conglomerate air bubbles, as described previously [9]. A 3D reconstruction of the dirty mass was obtained from the MDCT data using the Ziostation software (Ziosoft, Tokyo, Japan). As the mass could not be traced automatically by the software, tracing was done manually at 1 mm intervals on axial images, followed by 3D reconstruction (Fig. 1), and dirty mass volume (cm^3^) was then calculated using the software.

### Treatment

Following the confirmation of colorectal perforation intraoperatively, the surgical procedure was selected as follows. Resection of the diseased colonic segment with end colostomy Hartmann’s procedure (HP) was performed for perforation sites in the left-sided colon or rectum, or if the patient’s condition required a vasopressor agent during surgery. If the perforation site was right sided and the circulatory dynamics were stable, colectomy and primary anastomosis (PA) were performed. If the perforation site was unresectable due to cancer invasion or there was a relatively small perforation without severe inflammatory change around the perforation, stoma creation was performed. All the procedures were performed with open surgery and no laparoscopic surgeries were carried out. The open abdomen (OA) management was not performed in any patient due to lack of experience in our institute. Ventilation was continued after surgery until recovery from septic shock in the case of unstable circulatory condition, or if >50% oxygen concentration was required to maintain saturation of >90%. Polymyxin B-immobilized fiber direct hemoperfusion (PMX-DHP) was conducted when circulatory dynamics were unstable postoperatively even after the use of a vasopressor. Human recombinant thrombomodulin was administered if acute DIC score was ≥4 points.

### Statistical analysis

All analyses were conducted using the JMP 10 statistical software package (SAS Institute Inc., Tokyo, Japan). Receiver-operating characteristic (ROC) curve analysis was used to determine the cut-off point for dirty mass. Mann–Whitney *U* test was used for comparison of continuous variables. The chi-squared test was used to analyze the relationship of clinical characteristics. Multivariate analysis was conducted by a logistic regression analysis using factors with *p* value <0.01 in the univariate analysis. A *p* value <0.05 was considered to indicate statistically significant difference.

## Results

### Significance of dirty mass on prognosis

**Fig. 2:**
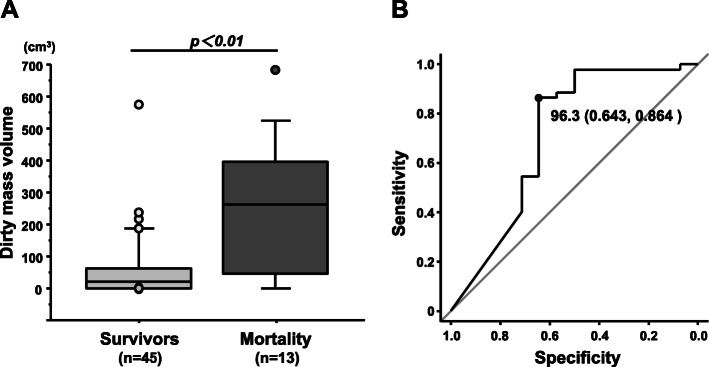
Comparison of dirty mass volume between the survivor and mortality groups. **a** Dirty mass volume was significantly larger in the mortality group than the survivor group. **b** ROC curve showed the highest peak at dirty mass volume of 96.3cm^3^, with 0.643 sensitivity and 0.864 specificity

Figure 1a and b shows manual tracing and 3D reconstruction of a dirty mass for volume calculation. The positional relationship between the dirty mass and the colorectum was easily recognized on the 3D image. Dirty mass was identified in 36/58 cases (62.1%); in all 36 cases, dirty mass was located next to perforated colorectum. Dirty mass volume was significantly larger in the mortality group than in the survivor group (Fig. 2a). The ROC curve showed the highest peak at volume of 96.3 cm^3^, with 0.643 sensitivity and 0.864 specificity (Fig. 2b).

### Factors of colorectal perforation predictive of mortality

Table 2 shows the comparison of patient background, physical examination, and disease factors between the survivor and mortality groups. The mortality group had significantly higher age, American Society of Anesthesiologists-physical status (ASA-PS) score frequency of preoperatively coexisting comorbidity and significantly lower mean arterial pressure (MAP) compared with the survivor group. Table 3 shows the comparison of therapeutic factors, examination data, and scores between the survivor and mortality groups. The mortality group had significantly higher frequency of ventilator use and thrombomodulin use and significantly higher dirty mass volume, DIC score, APACHE II score, SOFA score, and POSSUM score compared with the survivor group.

Univariate analysis revealed significant differences with *p*<0.01 for dirty mass volume, DIC score, APACHE II score, and SOFA score, and these four potential prognostic factors underwent further multivariate analysis. Logistic regression analysis revealed dirty mass volume and APACHE II score as independent prognostic factors, with significant difference, as shown in Table 4.

**Table 4:** Multivariate analysis of factors prognostic for colorectal perforation

	Odds ratio	*p* value
Dirty mass volume	1.010 (1.000-1.020)	0.006^**^
DIC score	1.310 (0.678-2.520)	0.424
APACHE II score	1.400 (1.070-1.850)	0.015^*^
SOFA score	1.170 (0.699-1.950)	0.554

Values in parentheses are the 95% confidence interval

*DIC* disseminated intravascular coagulation, *APACHE II* acute physiology and chronic health evaluation, *SOFA* sequential organ failure assessment, *POSSUM* physiological and operative severity score for the enumeration of mortality and morbidity

^*^p < 0.05, ^**^p < 0.01

### Stratification of mortality risk

**Fig. 3:**
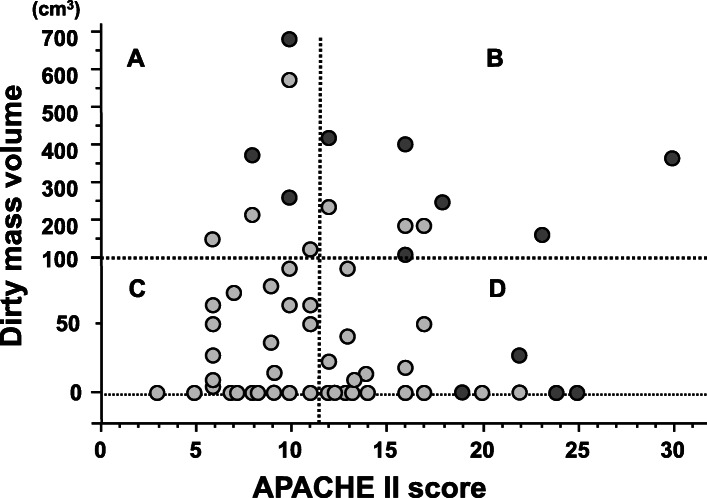
Stratification of mortality risk according to dirty mass volume and APACHE II score. Patients with colorectal perforation were divided into four groups (A to D) according to various combinations of dirty mass volume and APACHE II score. The mortality rates for groups A, B, C, and D were 42.8%, 66.6%, 0%, and 20.0%, respectively

Using the results of multivariate analysis, we divided the patients into four groups according to dirty mass volume and APACHE II score (Fig. 3): high volume/low score (group A), high volume/high score (group B), low volume/low score (group C), and low volume/high score (group D). Based on the ROC curve shown in Fig. 2b, a cut-off value of 100 was used for dirty mass volume, and the median APACHE II value of 11 was selected to assign high/low score. Mortality was 3/7 (42.8%) in group A, 6/9 (66.6%) in group B, 0/22 (0%) in group C, and 4/20 (20.0%) in group D. These results indicate that combined dirty mass volume and APACHE II score can be used to stratify the mortality risk of patients with colorectal perforation.

## Discussion

Dirty mass was initially reported by Saeki et al. as the CT finding of a focal collection of extraluminal fecal matter [9]. In their study, CT depicted this appearance in 15/29 cases (51.7%), size was variable (range, 1–6 cm), and dirty mass was located very close to the site of perforation. The present study is the first to undertake 3D reconstruction and to quantify dirty mass volume. 3D imaging enables surgeons to easily recognize the positional relationship between dirty mass and perforated colon and to plan the surgical procedure accurately. We identified dirty mass in 36/58 of the present cases (62.1%), all of which were located next to the perforated colorectum, consistent with the data published by Saeki et al. Furthermore, univariate and multivariate analysis revealed that dirty mass is a potential prognostic predictor.

In addition to dirty mass volume, the present univariate analysis identified the following as prognostic factors or scoring systems: older age, higher ASA-PS score, preoperative coexisting comorbidity, lower MAP, ventilator use, thrombomodulin use, higher DIC score, higher APACHE II score, higher SOFA score, and higher POSSUM score. Several predictive factors and scoring systems have been proposed for postoperative outcome in patients with colorectal perforation. Yamamoto et al. identified older age and low preoperative blood pressure as routinely available prognostic markers in colorectal perforation [4]. Harries et al. reported that preoperative ASA-PS and hemoglobin were significant independent predictors of mortality in perforated sigmoid diverticular disease [10], although there was no difference in hemoglobin levels between the present survivor and mortality groups. Scoring systems provide objective and systematic assessment of the severity of colorectal perforation, and various reports have described the utility of scoring systems for predicting the mortality risk of colorectal perforation. Nakamura et al. reported acute DIC score as the strongest predictor of mortality by multivariate analysis [11] and reported SOFA and POSSUM scores as significant markers in univariate analysis, but not APACHE II (*p* = 0.053). SOFA and POSSUM scores have been reported as useful prognostic markers for colorectal perforation in other studies [12, 13]. APACHE II is a disease-independent score for evaluation of highly dependent patients in the intensive care unit. Horiuchi et al. reported that APACHE II score was most strongly associated with poor prognosis, and reported mortality in >80% of patients with APACHE II score >15 and all patients with APACHE II score >20 [6]. In the present study, patients were divided according to APACHE II score at a median value of 11. When the patients were divided at different cut-off values, the mortality rate was 53% in patients with APACHE II score >15, and 83% in patients with APACHE II score >20 (Fig. 3), compatible with the findings of Horiuchi et al. Regarding ventilator and thrombomodulin use, these therapeutic choices were made in patients with severe conditions such as septic shock and DIC, which could have affected outcome in the mortality group.

Colorectal perforation followed by severe peritonitis requires emergency surgery such as HP and PA [14, 15]. There is no consensus regarding the most suitable surgical procedure and the indication for the procedure is commonly decided by the individual surgeon depending on the situation. In their propensity-score matched model, Tsuchiya et al. reported a higher rate of mortality for PA than for HP, and recommended that HP should be selected for patients with shock, immunosuppressive conditions, or advanced age [16]. Recently, less invasive approaches such as laparoscopic lavage have been reported as an effective alternative, although this procedure has a high rate of total reoperations and subsequent percutaneous drainage [17]. In the present study, OA was not performed. OA would be considered for the patients with severe condition because it may allow early identification and draining of residual infection, control any persistent source of infection, and remove more effectively infected or cytokine-loaded peritoneal fluid, preventing abdominal compartment syndrome and deferring definitive intervention [18, 19].

In the present study, mortality risk was stratified according to the combination of dirty mass volume and APACHE II score as an indicator of the severity of panperitonitis and the patient’s general condition. This stratification might enable surgeons to predict mortality risk and to select the better surgical approach, for example, to perform PA or even laparoscopic lavage for patients with low risk and OA for patients with high risk. Before being used in the clinical setting, the association of dirty mass volume and APACHEII score with the mortality should be validated and confirmed to be reproducible in another separate data series. Further prospective study is also required to evaluate the most appropriate therapeutic strategy.

## Conclusions

The findings of our study revealed the dirty mass volume as a potential prognostic indicator and that the combination of dirty mass volume and APACHE II score could stratify the postoperative mortality risk of patients with colorectal perforation. According to the risk stratification, surgeons might be able to decide the surgical procedures and could perform intensive postoperative management.

## Data Availability

The dataset supporting the conclusions of this article is included within the article and additional file.

## Abbreviations

CT: Computed tomography
MDCT: Multi-detector row computed tomography
ROC: Receiver-operating characteristic
DIC: Disseminated intravascular coagulation
APACHE II: Acute physiology and chronic health evaluation II
SOFA: Sequential organ failure assessment
POSSUM: Physiological and operative severity score for the enumeration of mortality and morbidity
HP: Hartmann’s procedure
PA: Primary anastomosis
OA: The open abdomen
PMX-DHP: Polymyxin B-immobilized fiber direct hemoperfusion
ASA-PS: American Society of Anesthesiologists-Physical Status
MAP: Mean arterial pressure
